# *QiHuangYiShen* Granules Modulate the Expression of LncRNA MALAT1 and Attenuate Epithelial-Mesenchymal Transition in Kidney of Diabetic Nephropathy Rats

**DOI:** 10.1155/2023/3357281

**Published:** 2023-01-31

**Authors:** Li-sha Yuan, Hua Du, Qiu-yue Ren, Rong-lu Yang, Shi-wei Liu, Shi-yi Liu, Kai-feng Shi, Bo Wang, Xiang-fei Meng, Tong-xia Li, Ning Zhang

**Affiliations:** ^1^Department of Endocrinology and Nephrology, Wangjing Hospital, China Academy of Chinese Medical Sciences, Beijing 100102, China; ^2^Beijing University of Chinese Medicine, Beijing 100029, China; ^3^Qingdao Traditional Chinese Medicine Hospital, Qingdao 266033, China

## Abstract

**Background:**

*QiHuangYiShen* granules (QHYS), a traditional Chinese herbal medicine formula, have been used in clinical practice for treating diabetic kidney disease for several years by our team. The efficacy of reducing proteinuria and delaying the decline of renal function of QHYS has been proved by our previous studies. However, the exact mechanism by which QHYS exerts its renoprotection remains largely unknown. Emerging evidence suggests that lncRNA MALAT1 is abnormally expressed in diabetic nephropathy (DN) and can attenuate renal fibrosis by modulating podocyte epithelial-mesenchymal transition (EMT).

**Objective:**

In the present study, we aimed to explore whether QHYS could modulate lncRNA MALAT1 expression and attenuate the podocyte EMT as well as the potential mechanism related to the Wnt/*β*-catenin signal pathway.

**Methods:**

SD rats were fed with the high-fat-high-sucrose diet for 8 weeks and thereafter administered with 30 mg/kg streptozotocin intraperitoneally to replicate the DN model. Quality control of QHYS was performed using high-performance liquid chromatography. QHYS were orally administered at 1.25, 2.5, and 5 g/kg doses, respectively, to the DN model rats for 12 weeks. Body weight, glycated haemoglobin, blood urea nitrogen, serum creatinine, 24-h proteinuria, and kidney index were measured. The morphologic pathology of the kidney was evaluated by Hematoxylin-eosin and Masson's trichrome staining. The expression level of lncRNA MALAT1 was determined by quantitative real-time polymerase chain reaction. In addition, the expression levels of podocyte EMT protein markers and Wnt/*β*-catenin pathway proteins in renal tissues were evaluated by Western blotting and immunohistochemistry.

**Results:**

The results showed that QHYS significantly reduced 24-h proteinuria, blood urea nitrogen, kidney index, and ameliorated glomerular hypertrophy and collagen fiber deposition in the kidney of DN rats. Importantly, QHYS significantly downregulated the expression level of lncRNA MALAT1, upregulated the expression of nephrin, the podocyte marker protein, downregulated the expression of desmin and FSP-1, and mesenchymal cell markers. Furthermore, QHYS significantly downregulated the expression levels of Wnt1, *β*-catenin, and active *β*-catenin.

**Conclusion:**

Conclusively, our study revealed that QHYS significantly reduced proteinuria, alleviated renal fibrosis, and attenuated the podocyte EMT in DN rats, which may be associated with the downregulation of lncRNA MALAT1 expression and inhibition of the Wnt/*β*-catenin pathway.

## 1. Introduction

Diabetic nephropathy (DN) is caused by diabetic microvascular damage, develops in approximately 40% of patients with diabetes mellitus, and is the leading cause of chronic kidney disease worldwide [1, 2]. Although tight control of blood glucose and blood pressure levels is helpful for the prevention of DN onset and progression, there is a large residual risk [3]. Therefore, an unmet need remains for innovative treatment strategies for preventing, treating, and reversing DN [3].

Long noncoding RNAs (LncRNAs) are defined as RNA transcripts longer than 200 nucleotides, which are recognized as playing crucial roles in numerous cellular processes [4]. Metastasis-associated lung adenocarcinoma transcript 1 (MALAT1, also called NEAT2), a ∼8 kb highly abundant lncRNA that is conserved across vertebrates and localizes to nuclear speckles, appears to orchestrate transcription, RNA processing, and other steps in gene expression by affecting nuclear architecture [5]. Accumulating evidence indicates that the expression of MALAT1 is dysregulated in DN and it plays an important role in promoting renal fibrosis in DN [6–8]. Recent studies have shown that lncRNA MALAT1 could interface with the Wnt/*β*-catenin pathway to enhance or repress podocyte epithelial-mesenchymal transition (EMT) [9].

Evidence has shown that the activation of the Wnt/*β*-catenin pathway mediated the podocyte EMT in DN, which was closely associated with the onset of proteinuria and renal fibrosis in DN patients [10–12]. The podocyte is a kind of terminally differentiated visceral epithelial cell. The transformation of podocyte to mesenchymal cell (EMT, also called podocyte dedifferentiation) is an important reversible mechanism leading to glomerulosclerosis [13]. EMT is a tightly regulated process where epithelial cells lose their epithelial features and gain the features of mesenchymal cells. For podocytes, the phenotype conversion occurs as follows: expressions of nephrin, podocin, P-cadherin, and ZO-1 are downregulated [14], while the expressions of desmin, vimentin, fibroblast-specific protein 1 (FSP-1), and *α*-smooth muscle actin (*α*-SMA) are upregulated [12].

*QiHuangYiShen* granules (QHYS) are formulated based on Traditional Chinese Medicine (TCM) theory. It has the effect of nourishing *qi* and *yin* and removing blood stasis. As a TCM formula, QHYS has been clinically used to treat DN for several years. Evidence from clinical random control trials and experiments of high-fat-high-sucrose diet/streptozotocin (HFSD/STZ)-induced DN model rats also displayed significant efficacy with reducing proteinuria and alleviating renal fibrosis [15–17]. However, the exact mechanism by which QHYS exerts its reno-protection remains largely unknown. It has been reported that *Astragaloside IV* (*AS-IV*), one of the active components of QHYS, could be involved in the modulation of EMT by affecting the expression levels of lncRNAs [18] and the activation of the Wnt/*β*-catenin signaling pathway [19].

Therefore, the present study aimed to explore whether QHYS could modulate lncRNA MALAT1 expression and attenuate the podocyte EMT as well as the potential mechanism related to the Wnt/*β*-catenin signal pathway.

## 2. Materials and Methods

### 2.1. Preparation of QHYS and Quality Control

QHYS (Patent No. CN104645132B) was extracted from five herbs: *Huangqi* (*Astragalus propinquus*), *Dihuang (radix rehmanniae recen)*, *Shanzhuyu (Cornus officinalis)*, *Danshen* (*Salvia miltiorrhiza*), and *Danggui (Angelica)*, in the ratio of 2 : 0.7 : 0.7 : 0.7 : 0.7. Quality control for the raw materials was performed according to the established guidelines in the Pharmacopoeia of The People's Republic of China, 2015. The herbs were prepared and standardized by Wangjing Hospital of China Academy of Chinese Medical Sciences. The daily volume for the adult is 24 g of granules. The granules were dissolved in distilled water (0.5 g/mL) for experimental use.

High-performance liquid chromatography (HPLC) was carried out to study the chemical fingerprint of QHYS. The process was as follows. QHYS decoction was filtered through a 0.45 *μ*m filter. HPLC was performed using an HPLC Agilent instrument (Agilent 1200, USA), with a Quaternary gradient pump (Agilent G 1211), autosampler (Agilent 1329A), Variable Wave Detector (VWD) (Agilent G1310), column heater system (Agilent 1316A), and Chromatography Workstation (Agilent 2170BA) with a mobile phase consisted of acetonitrile/water (gradient elution) at a flow rate of 1 mL/min. The column temperature was 30°C and the detection wavelength was set at 254 nm and 203 nm. A linear gradient elution was applied as follows: 2%–10% of acetonitrile from 0 to 10 minutes, 10%–20% of acetonitrile from 10 to 20 minutes, 20%–30% of acetonitrile from 20 to 30 minutes, 30%–60% of acetonitrile from 30 to 40 minutes, 60%–80% of acetonitrile from 40 to 50 minutes, 80%–95% of acetonitrile from 50 to 60 minutes, 95% of acetonitrile from 60 to 65 minutes.

**Figure 1:**
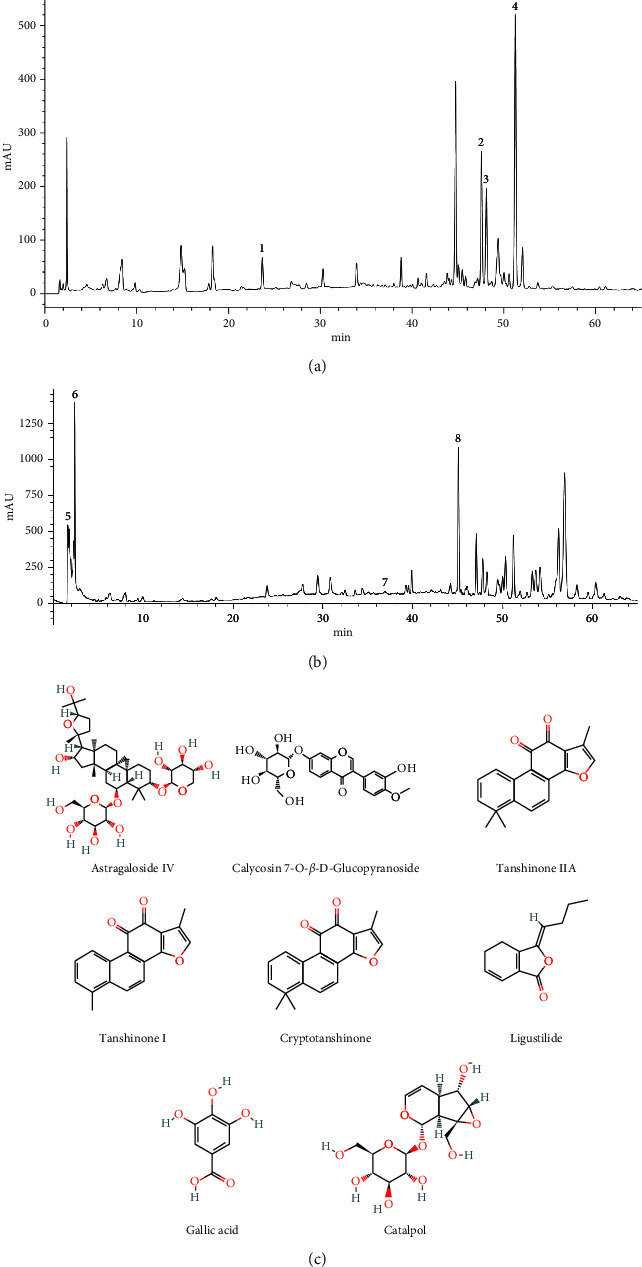
Analysis of the chemical constituents of QHYS by HPLC and the chemical structures of components. (a, b) HPLC chromatogram of QHYS and standard reference monitored at 254 nm and 203 nm. Peak 1: *Calycosin 7-O-β-D-Glucopyranoside*; peak 2: *Cryptotanshinone*; peak 3: *Tanshinone I*; peak 4: *Tanshinone IIA*; peak 5: *Gallic acid*; peak 6: *Catalpol*; peak 7: *Astragaloside IV*; peak 8: *Ligustilide*; (c) the chemical structures of the components.

Identification of HPLC peak fractions was carried out by comparing the retention time and UV spectra with the standards. Pure standards, from the National Institutes for Food and Drug Control (Beijing, China), including *Astragaloside IV* (110781-201717), *Calycosin 7-O-β-D-Glucopyranoside* (111920-201606), *Tanshinone IIA* (110766-201721), *Tanshinone I* (110867-201607), *Cryptotanshinone* (110852-201807), *Ligustilide* (111737-201608), *Gallic acid* (110831-201605), and *Catalpol* (110808-201711). These pure standards were used as external standards in the HPLC analysis. The resulting HPLC chromatogram of QHYS and the chemical structures of components were displayed in Figure 1.

### 2.2. Rat Models

The study protocol was approved by the Laboratory Animal Ethics Subcommittee of the Academic Committee of Beijing University of Chinese Medicine (Approval No. BUCM-4-2021092303-3174) and all experiments were performed in accordance with the Management Regulation of Beijing Laboratory Animal (2004 Revision).

Six-week-old male SD rats (160-200 g) were purchased from Beijing Vital River Laboratory Animal Technology Co., Ltd. (Beijing, China). All animals were kept under specific pathogen-free (SPF) conditions, with a 12-hlight-dark cycle at 23 ± 2°C and a humidity of 55 ± 5%. The animals were divided into two groups of feeding regimes: the first group was fed with a normal rat diet, while the other group was fed with HFSD [SPF (Beijing) Biotechnology Co., Ltd. (Beijing, China) (Production license number: SCXK (Beijing) 2019-0010)]. Both groups had free access to water. After eight weeks of HFSD, diabetes was induced with an intraperitoneal injection of freshly prepared STZ (30 mg/kg) dissolved in 0.1 M of citrate buffer (pH = 4.2). Three days after the STZ injection, fasting blood glucose levels in the tail vein were measured with the Roche ACCU-CHEK instant glucometer (AccuChek, Roche, Germany). Animals with blood sugar levels ≥300 mg/dL were considered diabetic and selected for further experiments. Body weight was measured every four weeks.

### 2.3. Experimental Design

The animals were randomly divided into six groups with six rats in each group: normal control group (NC) and diabetic control group (DC); the diabetic group was treated with 1.25 g/kg QHYS (D + QHYS-1.25); the diabetic group was treated with 2.5 g/kg QHYS (D + QHYS-2.5); the diabetic group was treated with 5 g/kg QHYS (D + QHYS-5); the diabetic group was treated with 0.01 g/kg losartan (D + L-0.01). The medium dose selection of 2.5 g/kg QHYS was calculated based on the human-rat equivalent dose ratio (1 : 6.25). QHYS and losartan were suspended in distilled water and delivered by oral gavage once daily for 12 weeks. At the same time, the rats in the NC and DC groups received the same volume of distilled water. After 12 weeks of treatment, 24-h urine collection was obtained by placing the rats in individual metabolic cages.

After 24-h urine collection, all rats were anesthetized after being fasted overnight and blood samples were collected. The serum was prepared by centrifuge at 3000 rpm for 15 min used for the analysis of serum creatinine and blood urea nitrogen (BUN), while the plasma was prepared by centrifuge at 2000 g for 15 min for the detection of glycated haemoglobin. The kidneys of each rat were dissected quickly, rinsed with normal saline, and weighed. The kidney index was calculated according to the formula as follows: kidney index = kidney weight (mg)/body weight (g) × 100%. One part of the kidney was stored in 4% paraformaldehyde for histological examination, while the other part was snap-frozen in liquid nitrogen and then stored at −80°C for further Western blot and quantitative Real-time polymerase chain reaction (qRT-PCR) tests. Finally, all rats were euthanized.

### 2.4. Biochemical Assays of Blood and Urine Samples

Biochemical parameters including BUN, serum creatinine, and 24-h proteinuria were measured using commercial kits from Roche Diagnostics (Shanghai) Co., Ltd. (Shanghai, China) by automatic biochemical analyzer cobas8000 (Roche, Germany). Glycated haemoglobin was detected using an enzyme-linked immunosorbent assay (ELISA) kit (Cloud-clone corp. Wuhan, China) according to the manufacturer's instructions.

### 2.5. Histopathology Analysis in Kidney

The kidneys were fixed in 4% paraformaldehyde and embedded in paraffin. Renal tissue sections (3-*μ*m thick) were stained with Hematoxylin-eosin (H&E) and Masson's trichrome reagent for analysis under light microscopy. The H&E stained sections were observed and the mean glomerular volume (GV) was calculated as follows: GV = *β*/*κ* × GA^3/2^, of which glomerular cross-sectional area (GA) was determined based on the average area of 10 glomeruli in each group (*n* = 6) [20, 21], *β* = 1.38, representing the shape coefficient of spheres (the idealized shape of glomeruli), and *κ* = 1.1, representing the size distribution coefficient. The Masson' trichrome stained sections were observed and quantification of collagen fiber positive area were performed in 6 random fields of each rat under ×800 magnification (*n* = 3). Semiquantitative analysis was carried out by Image J software (National Institutes of Health, Bethesda, MD, US).

### 2.6. Immunohistochemical Analysis

Immunohistochemistry (IHC) staining was performed according to the standard procedures of the immunohistochemistry kit. Briefly, the kidneys were fixed in 4% paraformaldehyde and embedded in paraffin. Paraffin sections were cut and mounted on slides. After microwave-based heating and antigen retrieval, the sections were stained with antibodies at 4°C overnight, followed by horseradish peroxidase-conjugated second antibodies at room temperature for 1h and observed under light microscopy. Primary antibodies and dilutions used were as follows: Nephrin (1 : 2000; Servicebio, GB11343, China); FSP-1 (1 : 500; Servicebio, GB11397, China). The IHC stained sections were observed, and the proportion of podocytes with positive expression of Nephrin and FSP-1 proteins in glomeruli was quantitatively detected under ×400 magnification (*n* = 3).

### 2.7. Western Blot

Collected kidney tissues were scratched with Radio Immunoprecipitation Assay (RIPA) Lysis Buffer (Beyotime Biotechnology, Shanghai, China) and centrifuged. The total protein was quantified with a BCA assay (Beyotime Biotechnology, Shanghai, China). 100 *μ*g of protein lysate were loaded and separated on 5%∼12% SDS-PAGE gel. After being transferred to a nitrocellulose membrane (Millipore, Darmstadt, Germany), the membranes were blocked and then incubated with primary antibodies overnight at 4°C, followed by incubation with suitable HRP-conjugated secondary antibodies (Beyotime Biotechnology, Shanghai, China) and were visualised using an enhanced chemiluminescence detection system (Tanon, Shanghai, China). *β*-actin was an equal loading control. Primary antibodies and dilutions used were as follows: Nephrin (1 : 1000; abcam, Ab216341, UK); Desmin (1 : 1000; abcam, ab32362, UK); FSP-1 (1 : 1000; CST, 24972, US); Wnt1 (1 : 500; Proteintech, 27935-1-AP, Wuhan, China); *β*-catenin (1 : 5000; Proteintech, 51067-2-AP, Wuhan, China); active *β*-catenin (1 : 1000; CST, 19807, US); *β*-actin (1 : 10000; Proteintech, 66009-1-Ig, Wuhan, China). The protein bands were quantified by densitometry using the Image J software. Experiments were performed in triplicate (*n* = 3).

### 2.8. Quantitative Real-Time Polymerase Chain Reaction (qRT-PCR)

Total RNA was extracted from renal cortical tissue using TRIzol reagent (TransGen Biotech Go., Ltd., Beijing, China) according to the manufacturer's instructions and was reversely transcribed to cDNA using the First Strand cDNA Synthesis Kit (Tiangen Biotech Co., Ltd., Beijing, China). The primers used for real-time PCR analysis are as follows: lncRNA MALAT1 (forward primer, 5′-GTTACCAGCCCAAACCTCAA-3′ and reversed primer, 5′-CTACATTCCCACCCAGCACT-3′),*β*-actin (forward primer, 5′-ACTGGGACGATATGGAGAAGA-3′ and reversed primer, 5′-AGCGCGTAACCCTCATAGAT-3′).*β*-actin was used as an endogenous control for mRNA. The qRT-PCR analysis was then performed using the real-time PCR kits (TransGen Biotech Go., Ltd., Beijing, China) and a 7500 H T Fast Real-Time PCR System (ABI). Experiments were performed in triplicate of each rat (*n* = 3).

### 2.9. Statistical Analysis

All data were expressed as the mean ± SEM. The differences between groups were analyzed by one-way ANOVA followed by a multiple comparisons test. All experiments have repeated a minimum of three times, and representative experiments are shown. Statistical difference was considered when *p* < 0.05. All statistical analysis was performed using GraphPad Prism 7.0 Software (La Jolla, CA, US).

## 3. Results

### 3.1. Effect of QHYS on Body Weight and Glycated Haemoglobin Levels in Different Groups

**Figure 2:**
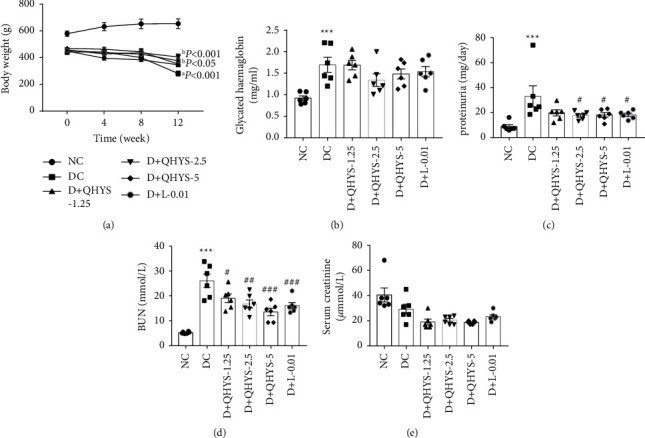
Comparison of body weight, glycated haemoglobin, 24-h proteinuria, serum BUN, and serum creatinine among different groups. (a) Time curves of body weight in different groups; (b) plasma levels of glycated haemoglobin; (c) 24-h proteinuria; (d) serum BUN; (e) serum creatinine. Data are expressed as the mean ± SEM (*n* = 6). ^a^*P* < 0.001 vs. NC group at all time points; ^b^*P* < 0.001 vs. DC group at the last time point; ^b^*P* < 0.05 vs. DC group at the last time point. ^*∗∗∗*^*P* < 0.001 vs. NC group. ^#^*P* < 0.05 vs. DC group. ^##^*P* < 0.01 vs. DC group. ^###^*P* < 0.001 vs. DC group.

The persistent lower body weight and higher glycated haemoglobin levels over the 12-week intervention period were displayed in the DC group compared with the NC group (Figures 2(a) and 2(b)). At the 12-week time point, body weights in the D + QHYS-2.5 group and D + L-0.01 group were significantly increased when compared with the DC group (Figure 2(a)). There was no significant difference in the glycated haemoglobin levels between the QHYS treatment groups and the DC group (Figure 2(b)).

### 3.2. QHYS Treatment Reduced the 24-h Proteinuria and Serum BUN Levels of DN Rats

The 24-h proteinuria and serum BUN levels in the DC group were observed to be significantly higher than those in the NC group. Compared with the DC group, those indexes in the D + QHYS-2.5, D + QHYS-5, and D + L-0.01 groups were significantly decreased to varying degrees (Figures 2(c) and 2(d)). However, the serum creatinine level was lower in the DC group compared with the NC group (Figure 2(e)). Considering the potential effect of body weight, the serum creatinine of the normal rats with similar body weight to those in the DC group was detected additionally, and the result showed that the rats in the DC group had higher serum creatinine when compared with the normal rats (Supplementary data Figure S1). Nevertheless, there was no significant difference in serum creatinine between DC and QHYS treatment rats.

### 3.3. QHYS Treatment Ameliorated Renal Pathological Damages in DN Rats

**Figure 3:**
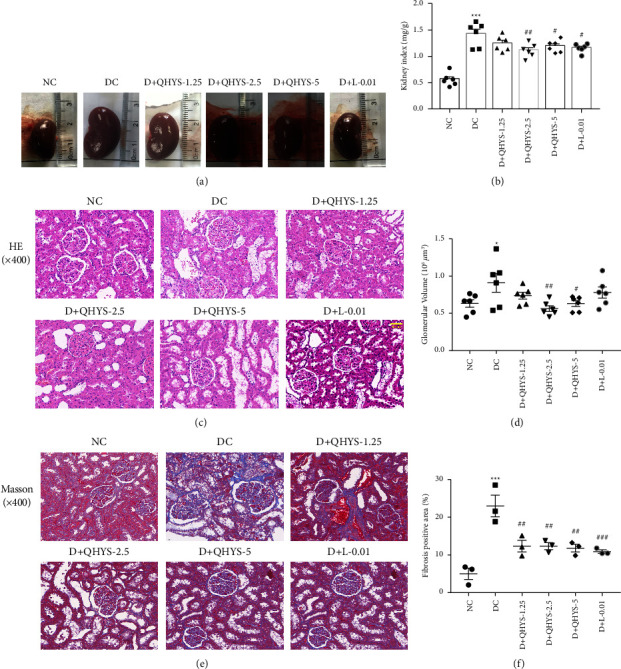
Morphological changes of the kidney in different groups. (a) Representative images of the kidneys; (b) the kidney index; (c) representative images of H&E staining; (d) quantification of glomerular volume; (e) Masson's trichrome staining showing the glomerular and tubulointerstitium fibrosis of renal sections; (f) quantification of collagen fiber positive area. ^*∗*^*P* < 0.05 vs. NC group. ^*∗∗∗*^*P* < 0.001 vs. NC group. ^#^*P* < 0.05 vs. DC group. ^##^*P* < 0.01 vs. DC group. ^###^*P* < 0.001 vs. DC group.

As shown in Figure 3, the kidneys of the rats in the DC group were apparently enlarged compared with those in the NC group. QHYS and losartan treatment reduced the apparent size of the kidneys (Figure 3(a)). At the same time, the change of kidney mass was corresponded with that of the kidney apparent size (Figure 3(b)). H&E staining images showed that rats treated with QHYS had a significant decrease in glomerular hypertrophy and glomerular volume when compared with the DC group (Figures 3(c) and 3(d)). The glomerulosclerosis and tubulointerstitial fibrosis showed by Masson's trichrome staining were increased in the DC group relative to the NC group, whereas the collagen fiber deposition was significantly alleviated after a 12-week treatment with QHYS (Figures 3(e) and 3(f)).

### 3.4. QHYS Reduced the Level of lncRNA MALAT1 in the Kidney of DN Rats

**Figure 4:**
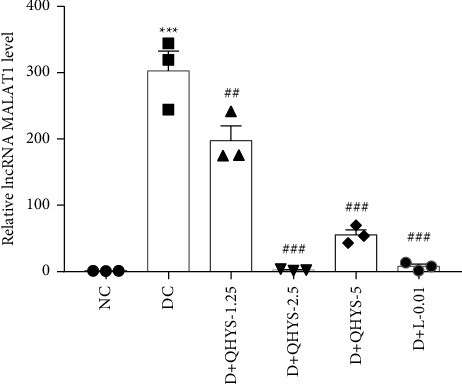
The relative level of lncRNA MALAT1 based on qRT-PCR. ^*∗∗∗*^*P* < 0.001 vs. NC group. ^###^*P* < 0.001 vs. DC group.

To figure out whether QHYS can regulate the lncRNA MALAT1 expression level, we detected the expression of MALAT1 in the renal cortex by qRT-PCR. The results showed that the level of lncRNA MALAT1 was significantly increased in the DC group compared with the NC group. When DN rats were treated with QHYS, the MALAT1 expression levels were lower than those in DN rats without treatment (Figure 4).

### 3.5. QHYS Treatment Attenuated Renal Epithelial-Mesenchymal Transition in the Kidney of DN Rats

**Figure 5:**
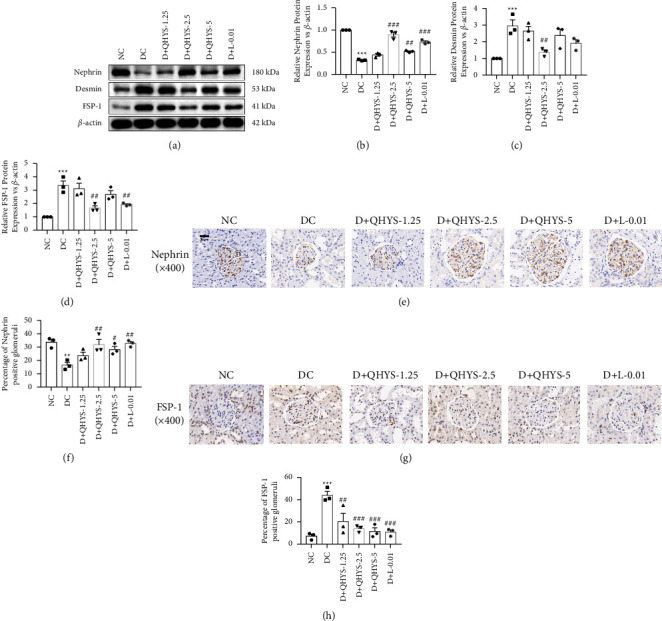
The relative expressions of nephrin, desmin, and FSP-1 in the renal cortex of rats in different groups. (a) Representative images of western blot of nephrin, desmin, and FSP-1; (b–d) semiquantitative analysis of the relative expressions of nephrin, desmin, and FSP-1 proteins; (e) representative images of nephrin IHC staining; (f) semiquantitative analysis of the relative expression of nephrin. (g) Representative images of FSP-1 IHC staining. (h) Semiquantitative analysis of the relative expression of FSP-1. ^*∗∗∗*^*P* < 0.001 vs. NC group. ^#^*P* < 0.05 vs. DC group. ^##^*P* < 0.01 vs. DC group. ^###^*P* < 0.001 vs. DC group.

We next detected the expression levels of markers related to EMT, such as nephrin, FSP-1, and desmin. The Western blot results showed that the nephrin expression level was significantly downregulated, but the expression levels of desmin and FSP-1 were upregulated in the renal cortex of rats in the DC group compared with the NC group. QHYS treatment partially inhibited the FSP-1 and desmin expression levels and recovered the nephrin expression in DN rats (Figures 5(a) and 5(d)). Correspondingly, immunohistochemistry showed that the expression level of nephrin in the glomerulus was downregulated, but the FSP-1 was upregulated in the DC group compared with the NC group. These changes were reversed by the treatment with QHYS (Figures 5(e)–5(h)).

### 3.6. QHYS Regulated the Wnt/*β*-Catenin Signaling Pathway in the Kidney of DN Rats

**Figure 6:**
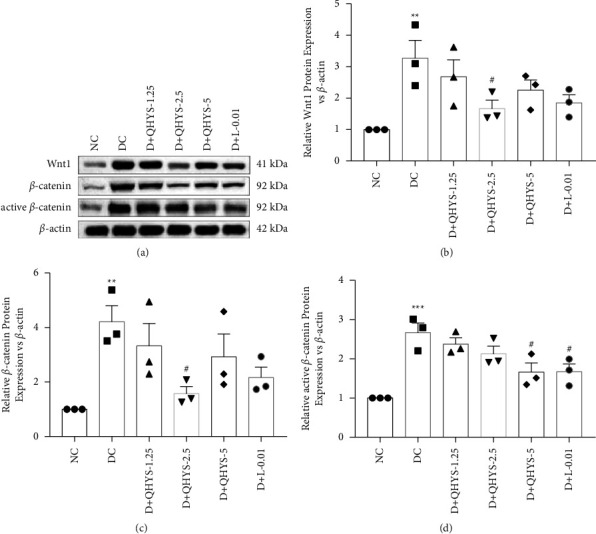
The relative expressions of Wnt1, *β*-catenin, and active *β*-catenin in the renal cortex of rats in different groups. (a) Representative images of western blot of Wnt1, *β*-catenin, and active *β*-catenin; (b–d) semiquantitative analysis of the relative expressions of Wnt1, *β*-catenin, and active *β*-catenin. ^*∗∗*^*P* < 0.01 vs. NC group. ^*∗∗∗*^*P* < 0.001 vs. NC group. ^#^*P* < 0.05 vs. DC group.

To further research the potential molecular mechanisms regarding the anti-EMT effects of QHYS in *vivo*, the levels of markers in the Wnt/*β*-catenin pathway were examined. As the Western blot showed, the expression levels of Wnt1, *β*-catenin, and active *β-*catenin were significantly increased in the DC group when compared with the NC group, indicating that the Wnt/*β-*catenin signaling pathway in the kidney of DN rats was activated. With the QHYS intervention, the Wnt1, *β*-catenin, and active *β*-catenin expression levels were significantly decreased (Figures 6(a)–6(d)).

## 4. Discussion

The present study was designed to explore the effect on regulating lncRNA MALAT1 expression and attenuating podocyte EMT of QHYS in DN rats. We constructed a DN rat model induced by HFSD combined with low-dose STZ injection. The model manifests as high glucose, weight loss, increased urinary protein excretion, and renal tissue-damaging effects that include increases in kidney mass, glomerular hypertrophy, and glomerulosclerosis [22–25]. We investigated the pharmacodynamic effects of QHYS on the DN model rats. Our study showed that treatment with QHYS significantly reduced proteinuria, BUN, kidney hypertrophy, and ameliorated the glomerular hypertrophy and glomerulosclerosis observed in DN model rats, while the serum creatinine and glycated haemoglobin levels were not affected, which are consistent with previous studies by our teams [16, 17].

LncRNA MALAT1, one of the earliest lncRNAs to be described in the process of cancer metastasis, can affect cancer invasion and metastasis by regulating EMT [26]. It was reported that MALAT1 was highly expressed in diabetic kidney disease (DKD) patients [6] and was upregulated in renal tissues of STZ-induced diabetes rats. Knockdown of MALAT1 in STZ-induced rats improved the kidney pathology and inhibited the expressions of col I, col IV, FN, and LN in renal tissues, which demonstrated that MALAT1 may be related to renal fibrosis [27].

Podocyte EMT, as a major source of myofibroblasts, is closely associated with the progress of the renal fibrosis in DN [13]. The activation of the Wnt/*β*-catenin signaling pathway, induced by high glucose, has been proven to mediate the podocyte EMT [28]. In the canonical Wnt/*β*-catenin pathway, *β*-catenin is inhibited by the Axin complex in the cytoplasm without the Wnt signaling factor. In contrast, when Wnt signaling is activated by high glucose, active *β*-catenin, the dephosphorylated form of *β*-catenin, accumulates in the cytoplasm and then transfers to the nucleus, in where it activates Wnt target genes to cause podocyte EMT [14]. Studies showed that Wnt1 protein and nuclear localization of active *β*-catenin was induced in the podocytes of human kidney biopsies from patients with DN [29]. Overexpression of Wnt1 in *vivo* activated glomerular *β*-catenin and aggravated albuminuria, whereas blockade of Wnt signaling ameliorated podocyte lesions [29]. Moreover, many studies have found that MALAT1 can mediate the conduction of the Wnt/*β*-catenin signaling pathway and be involved in the regulation of podocyte EMT [9, 30]. MALAT1 knock-down partially reversed podocyte damage caused by high glucose, and inhibited the downregulation of nephrin expression and upregulation of desmin expression, and abolished the upregulation of *β*-catenin expression [8, 9].

Results from the present study showed that the level of lncRNA MALAT1 was upregulated in DN model rats and was downregulated by treatment with QHYS, suggesting that QHYS could restore abnormally elevated lncRNA MALAT1 in the kidney of DN rats. Moreover, it was observed that nephrin expression was downregulated and the expression levels of desmin and FSP-1 were upregulated in the renal cortex of DN rats, which was consistent with previous studies [31, 32]. Treatment with QHYS reversed these changes, indicating that QHYS could alleviate podocyte EMT in DN rats. Furthermore, the present study showed that the expression levels of Wnt1, *β*-catenin, and active *β*-catenin were upregulated in DN model rats, whereas the changes were inhibited by the treatment with QHYS, demonstrating that QHYS could inhibit the activation of the Wnt/*β*-catenin signaling pathway. To sum up, the regulation of lncRNA MALAT1 by QHYS may be a potential mechanism for its effect on attenuating renal fibrosis and podocyte EMT.

*AS-IV*, one of the main ingredients of QHYS, is a bioactive saponin extracted from the *Astragalus* root. Both *in vivo* and *in vitro* experiments confirmed that *AS-IV* had an anti-EMT effect, was capable of inhibiting the Wnt/*β*-catenin pathway, and ultimately ameliorated the renal injury caused by high glucose [19]. Evidences showed that *AS-IV* could inhibit podocyte dedifferentiation, increased nephrin expression level, and decreased the *α*-SMA expression level in podocytes by mediating the Wnt/*β*-catenin pathway [33]. *Tanshinone IIA* (*Tan IIA*), a compound extracted from *Salvia miltiorrhiza,* was also found in QHYS through HPLC. It could reduce the BUN level and collagen formation in the renal tissues of STZ-induced diabetic rats [34]. *Gallic acid*, another compound of QHYS, could lower serum creatinine and BUN levels, improve body weight, and decrease kidney hypertrophy [35].

However, some limitations were present in our study. Although we confirmed that QHYS could reverse the changes of lncRNA MALAT1 expression and podocyte EMT markers in DN rats; we did not use gene-knockout animal models, neither give evidence in *vitro*. Further experiments in MALAT1 overexpression or suppression models and in *vitro* are needed to support the conclusion.

## 5. Conclusion

In conclusion, the present study found that QHYS could reduce proteinuria excretion, and improve the renal histopathological morphology, and attenuate podocyte EMT in DN model rats, probably by downregulating lncRNA MALAT1 expression and inhibiting the Wnt/*β*-catenin signaling pathway.

## Data Availability

The original data used to support the findings of this study are available from the corresponding author upon request.

## Supplementary Materials

Supplementary Materials The following is the supplementary data related to this article (Figure S1).
