# Supplementary material for: QiHuangYiShen Granules Modulate the Expression of LncRNA MALAT1 and Attenuate Epithelial-Mesenchymal Transition in Kidney of Diabetic Nephropathy Rats
Source: Evid Based Complement Alternat Med. 2023 Jan 31;2023:3357281. doi: 10.1155/2023/3357281 (PMC9904933; doi:10.1155/2023/3357281)

**Supplementary Data**

**Figure S1. Comparison of body weight and serum creatinine between rats in DC group and normal rats.** (A) Body wight. (B) Serum creatinine. Data are expressed as the mean ± SEM (*n*=6). **P*<0.05 vs. normal rats.

1. (B)


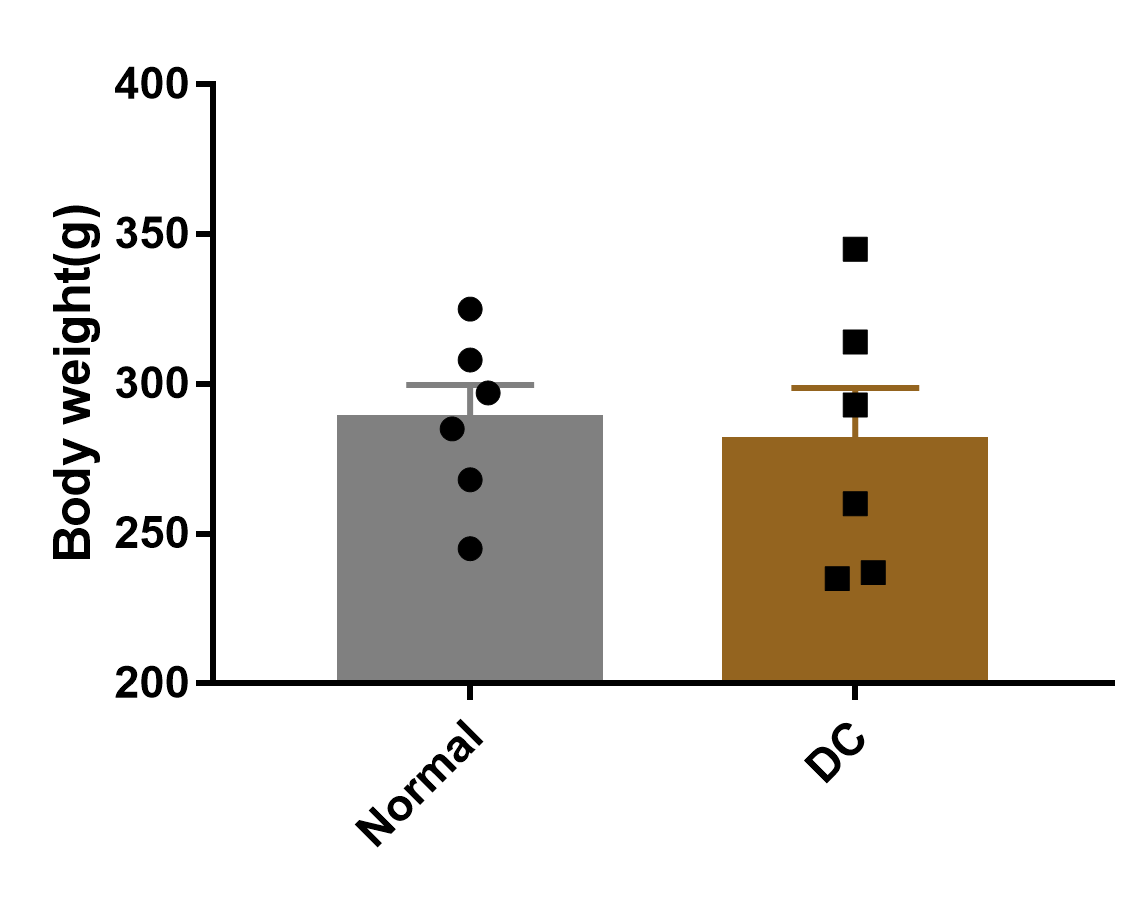

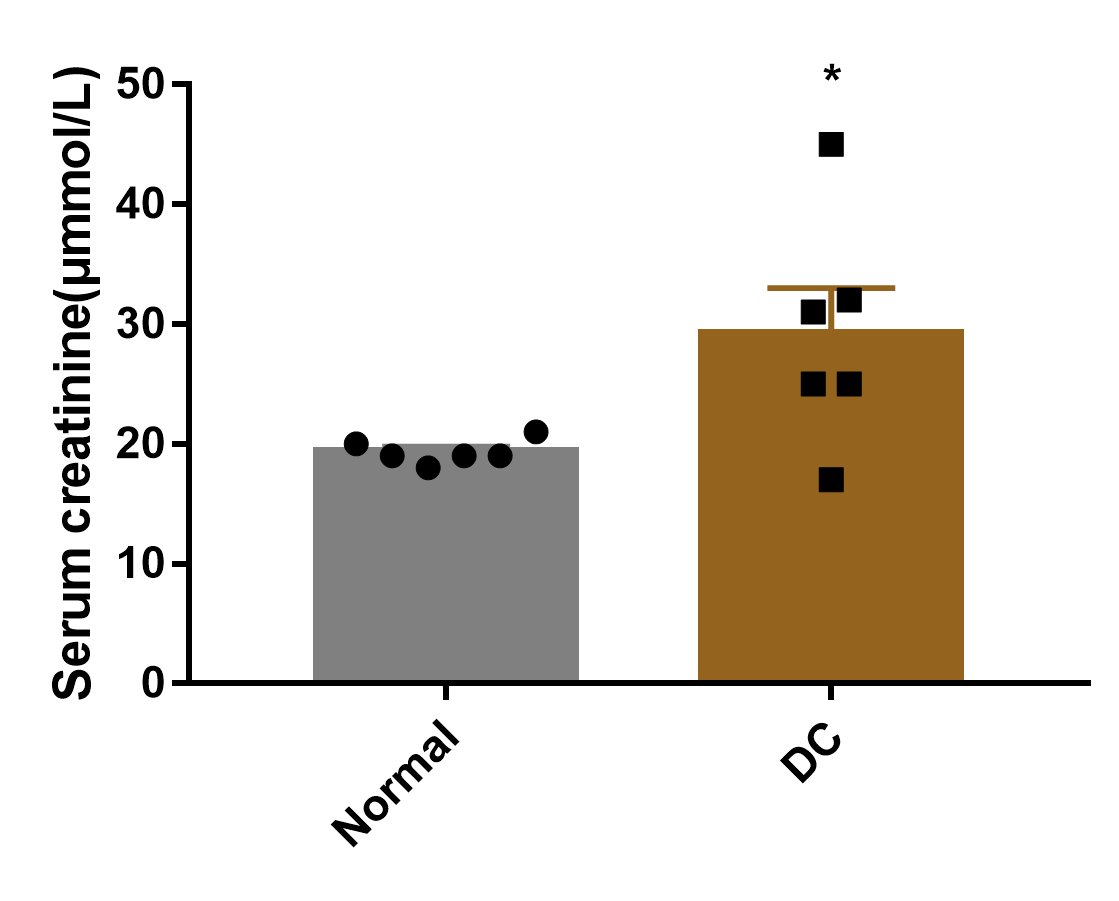

Supplement: Supplementary Materials — The following is the supplementary data related to this article (Figure S1). [file 3357281.f1.docx]
